# The *Pseudomonas aeruginosa rhlG* and *rhlAB* genes are inversely regulated and RhlG is not required for rhamnolipid synthesis

**DOI:** 10.1186/1471-2180-14-160

**Published:** 2014-06-19

**Authors:** Alexis Bazire, Alain Dufour

**Affiliations:** 1 Université de Bretagne-Sud, EA 3884, LBCM, IUEM, F-56100 Lorient, France

**Keywords:** RhlG, Rhamnolipid, *Pseudomonas aeruginosa*, AlgU

## Abstract

**Background:**

*Pseudomonas aeruginosa* produces rhamnolipid biosurfactants involved in numerous phenomena including virulence. The transcriptional study of the *rhlAB* operon encoding two key enzymes for rhamnolipid synthesis led to the discovery of the quorum sensing system RhlRI. The latter positively controls the transcription of *rhlAB*, as well as of *rhlC*, which is required for di-rhamnolipid synthesis. The *rhlG* gene encodes an NADPH-dependent β-ketoacyl reductase. Although it was reported to be required for the biosynthesis of the fatty acid part of rhamnolipids, its function in rhamnolipid synthesis was later questioned. The *rhlG* transcription and its role in rhamnolipid production were investigated here.

**Results:**

Using 5′-RACE PCR, a *luxCDABE*-based transcriptional fusion, and quantitative reverse transcription-PCR, we confirmed two previously identified σ^70^- and σ^54^-dependent promoters and we identified a third promoter recognized by the extra-cytoplasmic function sigma factor AlgU. *rhlG* was inversely regulated compared to *rhlAB* and *rhlC*: the *rhlG* transcription was down-regulated in response to *N*-butyryl-l-homoserine lactone, the communication molecule of the RhlRI system, and was induced by hyperosmotic stress in an AlgU-dependent manner. Consistently with this transcriptional pattern, the single or double deletions of *rhlG* and PA3388, which forms an operon with *rhlG*, did not dramatically impair rhamnolipid synthesis.

**Conclusion:**

This first detailed study of *rhlG* transcription reveals a complex regulation involving three sigma factors and *N*-butyryl-l-homoserine lactone. We furthermore present evidences that RhlG does not play a key role in rhamnolipid synthesis.

## Background

*Pseudomonas aeruginosa* is a ubiquitous Gram negative bacterium and an opportunistic human pathogen, in particular responsible for the chronic lung infection of cystic fibrosis patients. *P. aeruginosa* produces rhamnolipids, which are glycolipidic biosurfactants consisting of one or two hydrophilic l-rhamnose molecules (mono- and di-rhamnolipids, respectively) and of a hydrophobic fatty acid moiety, see [1] for review. Rhamnolipids are involved in a number of functions, such as the uptake of poorly soluble substrates, surface motility, biofilm development, or interaction with the immune system [2], and are considered as virulence factors. Most of the rhamnolipid biosynthetic pathway is clearly established [1,3]: RmlA, RmlB, RmlC, and RmlD are responsible for dTDP-l-rhamnose synthesis from glucose-1-phosphate, while RhlA supplies the acyl moieties by converting two molecules of β-hydroxylacyl-Acyl Carrier Protein (ACP) in one molecule of β-D-(β-D-hydroxyalkanoyloxy) alkanoic acid (HAA). Finally, the rhamnosyltransferase RhlB links one l-rhamnose molecule to one HAA to yield one mono-rhamnolipid, which either will be the final product or will be the substrate of the second rhamnosyltransferase RhlC to obtain one di-rhamnolipid. RhlG was described as an NADPH-dependent β-ketoacyl reductase specifically involved in rhamnolipid synthesis [4]. It was proposed to work just upstream of RhlA, converting one β-ketoacyl-ACP molecule in one β-hydroxylacyl-ACP [5]. These conclusions were based on: i) the amino acid sequence similarities between RhlG and FabG, which is part of the general fatty acid synthetic pathway; ii) the absence of rhamnolipid production by an *rhlG* mutant of *P. aeruginosa* PAO1; and iii) similarities between the promoters of the *rhlG* gene and of the *rhlAB* operon, suggesting a coordinated expression of the genes involved in rhamnolipid synthesis [4]. However, two subsequent articles questioned the RhlG function. A structural and biochemical study of RhlG confirmed that it is an NADPH-dependent β-ketoacyl reductase, but indicated that the RhlG substrates are not carried by the ACP [6]. Zhu and Rock [3] then reported that RhlG was not required for rhamnolipid synthesis in the heterologous host *Escherichia coli* and that *rhlG* mutants of *P. aeruginosa* PA14 and PAO1 were not affected in rhamnolipid production. These authors concluded that RhlG plays no role in rhamnolipid formation and that its physiological substrate remains to be identified [3]. The transcriptional regulation of the *rhlG* gene has not been so far studied in more details than in [4]. Among the rhamnolipid-related genes, the *rhlAB* operon was the first and most extensively studied at the transcription level. These works led to the discovery of the RhlRI quorum sensing (QS) system, which is encoded by genes lying just downstream of *rhlAB* and is required for *rhlAB* transcription [7-10]. RhlRI is a LuxRI-type QS system [11], RhlI synthesizing the communication molecule *N*-butyryl-l-homoserine lactone (C_4_-HSL) which binds to the transcription regulator RhlR. Medina *et al*. [12] showed that RhlR directly binds to a specific DNA sequence upstream of *rhlA*, regardless of the presence or not of C_4_-HSL. Without C_4_-HSL, RhlR would act as a transcriptional repressor of *rhlAB*, whereas RhlR/C_4_-HSL would activate transcription. It should be noted that the RhlRI system is embedded within a complex QS network including the LasRI system with its autoinducer *N*-(3-oxododecanoyl)-l-homoserine lactone (3OC_12_-HSL) and the *Pseudomonas* Quinolone Signal (PQS) system [13,14], but RhlR is the main direct QS regulator of *rhlAB* transcription [1]. A single transcription start site identified upstream of *rhlA* could result from two putative promoters, one of which would dependent on the alternative sigma factor σ^54^ (RpoN) and the other on the primary sigma factor σ^70^[7]. Rhamnolipid production was indeed impaired in *rpoN* mutants [7,8], but subsequent data showed that the RhlR/C_4_-HSL complex activates the *rhlA* promoter independently from σ^54^[12] and it remains unclear if the latter acts only indirectly on *rhlAB* transcription. Determining the 5′ end of *rhlG* mRNAs by primer extension led to the identification of two overlapping promoters likely dependent on the sigma factors σ^70^ and σ^54^[4]. These promoters are preceded by a putative “lux box” which could be a LasR and/or RhlR target sequence [4]. Since the *rhlG* mRNA concentration was only slightly lower in a *lasR* mutant than in the wildtype strain, it was concluded that LasR is not a direct activator of *rhlG* transcription, but it remained possible that RhlR plays this role [4]. *rhlG* was thus proposed to be regulated similarly as the *rhlAB* operon [4], consistently with the notion that the encoded enzymes belong to the same biosynthesis pathway. It turned out later that the transcription of the PA1131-*rhlC* and the *rmlBDAC* operons is also mainly dependent on RhlR/C_4_-HSL, and the PA1131-*rhlC* promoter was proposed to be σ^54^-dependent [15,16].

In previous works, we examined the effect of hyperosmotic stress on rhamnolipid production, accumulation of QS communications molecules, and expression levels of related key genes [17,18]. We observed that hyperosmotic condition led to down-regulations of *rhlAB* and *rhlC* and prevented rhamnolipid production. These works prompted us to investigate in more details the transcriptional regulation of *rhlG* and to compare its transcription pattern to the *rhlAB* and *rhlC* ones. Here, we mapped the *rhlG* promoters, confirming that the σ^70^-dependent promoter is functional and identifying a third promoter dependent on the alternative sigma factor AlgU. On the contrary to *rhlAB* and *rhlC*, *rhlG* was down-regulated by quorum sensing and induced under hyperosmotic stress. We constructed single PAO1 mutants with deletions in *rhlG* or PA3388 (which is co-transcribed with *rhlG*), and the double *rhlG*/PA3388 mutant. The phenotypes of the mutants confirmed that RhlG is not involved in rhamnolipid biosynthesis.

## Results

### *rhlG* transcription is dependent on three sigma factors: σ^70^, AlgU and σ^54^

**Figure 1:**
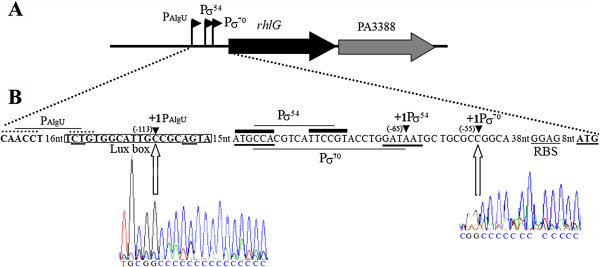
**Promoter mapping of *rhlG. *A**: Schematic representation of the *rhlG* locus. Black flags indicate the promoters P_AlgU_, Pσ^54^, and Pσ^70^; and arrows indicate the *rhlG* and PA3388 genes. **B**: Annotated sequence of the *rhlG* promoter region. Black triangles indicate the three transcription start sites (+1) and the negative numbers provide their position relative to the *rhlG* translation initiation codon. The promoter sequences recognized by the sigma factors AlgU, σ^54^, and σ^70^ are respectively point over lined, full trait over lined, and underlined. The “lux box” as proposed in [4] is boxed with the two highly conserved dinucleotides underlined. The chromatograms show the results of 5′-RACE PCR allowing us to identify the major transcription start sites resulting from P_AlgU_ and the minor from 1 Pσ^70^, the white arrow corresponding to the last base before the polyC tail added to the 5′ extremity of cDNA. The transcription start site resulting from Pσ^54^ was identified in [4].

We used 5′-RACE PCR to determine which promoter mainly controls *rhlG* transcription when cells were grown during 24 h in PPGAS medium, which favors rhamnolipid production [19]. We used the *P. aeruginosa* PAO1 strain containing pAB134, which carries the *luxCDABE* operon under the control of the *rhlG* promoter region (*prrhlG*), extending from − 413 to −23 relative to the first base of the *rhlG* translation initiation codon. We chose this strain since the multi-copy pAB134 plasmid led to higher amounts of mRNAs than the genomic mono-copy *rhlG* gene, thereby facilitating the experiment. Three internal *luxCDABE* primers were used to synthesize cDNAs and amplify them by PCR. A mix of two DNA fragments, both of ~ 400 pb was obtained after the last PCR. They were sequenced, identifying two different transcription start sites at positions −113 and −55 relative to the *rhlG* translation initiation codon (Figure 1). The weakest signal (−55) corresponded to the transcription start site previously identified by Campos Garcia et *al.*[4] as arising from a σ^70^-dependent promoter. The strongest signal (−113) revealed a novel transcription start site preceded by the sequence CAACCT − N_16_ − TCTG, which is similar to the consensus sequence for AlgU-dependent promoters, GAACTT − N_16–17_ − TCTG [20]. AlgU is the extra-cytoplasmic function (ECF) sigma factor involved in alginate overproduction leading to mucoidy, response to some stresses, and biofilm stability [21-23].

**Figure 2:**
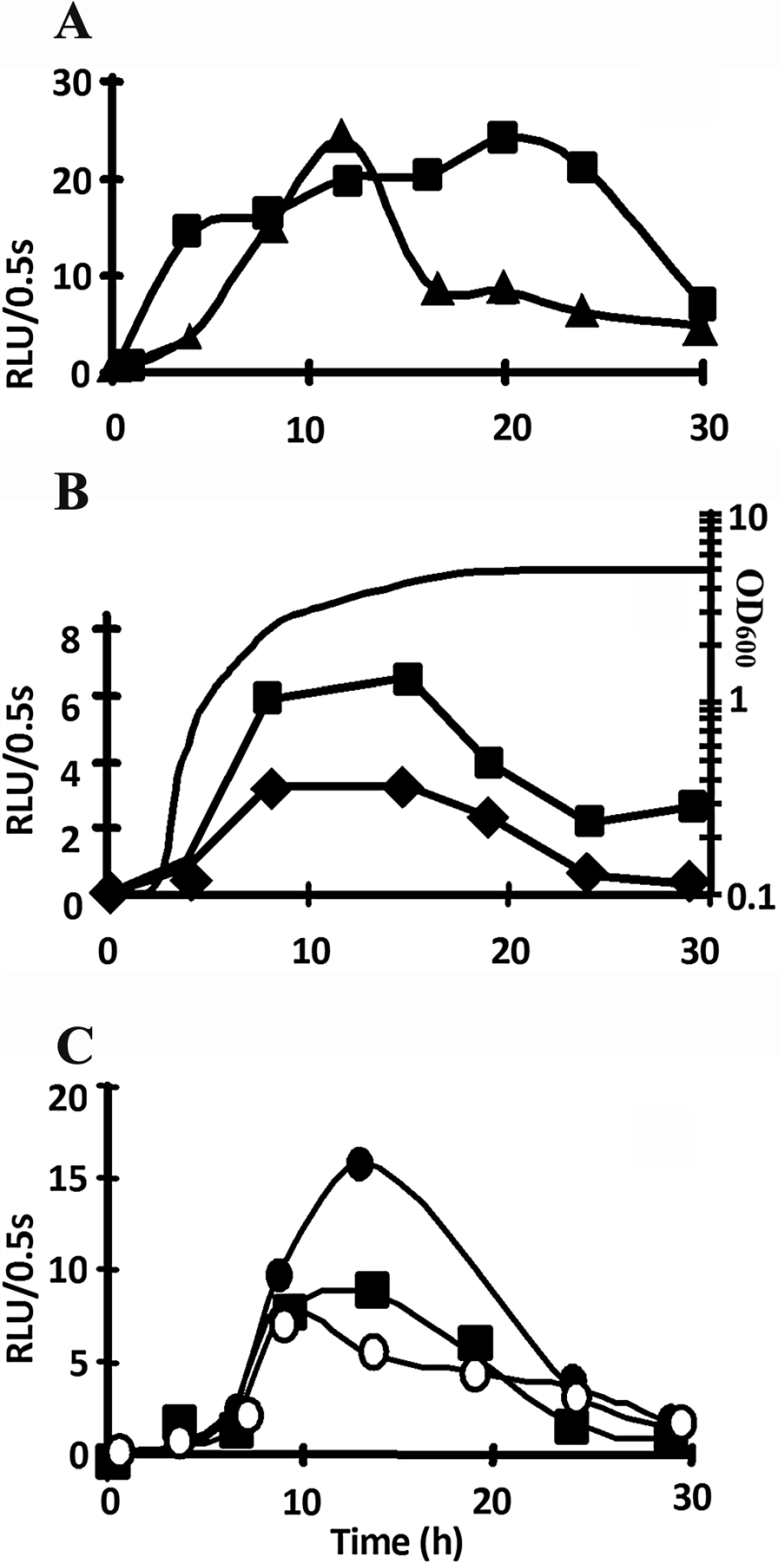
**Transcriptional activity of *prrhlG*.** Promoter activity was followed by measuring the luminescence from *P. aeruginosa* PAO1 wildtype (squares) and mutant strains, harbouring pAB134, which contains the *prrhlG*::*luxCDABE* transcriptional fusion. Activity was compared between the wildtype PAO1 strain and PAOU (*algU* mutant, triangles) **(A)**; PAO1 and PAO6358 (*rpoN* mutant, diamonds) **(B)**, and PAO1 and PDO100 (*rhlI* mutant) strain complemented with C_4_-HSL (open circles) or not (blacks circles) **(C)**. Activity is expressed in Relative Units of Luminescence per 0.5 second in function of time growth. Gain for luminescence detection was automatically set for each experiment. Results are representative of 2 complete experiments and of several additional experiments with fewer time points, standard deviations were < 6% for all values. Curve without symbol in panel B: growth curve of PAO1.

The pAB134 plasmid was primarily constructed to quantify the *prrhlG* activity in the course of bacterial growth by measuring the luminescence resulting from the LuxCDABE proteins. To verify the role of AlgU in the transcription of *rhlG*, *P. aeruginosa* PAO1 and its *algU* mutant strain PAOU [21] were transformed by pAB133 (containing the promoter-less *luxCDABE* operon, used to quantify the luminescence baseline) and pAB134. Strains were grown in PPGAS medium and luminescence was followed during 30 h. Figure 2A shows that the *prrhlG* activity was ~3-fold lower in *P. aeruginosa* PAOU than in PAO1 during stationary phase (from 16 h of growth, a typical growth curve is shown on Figure 2B). To ascertain that the results were not biased by the reporter gene and/or vector, we assayed *rhlG* mRNA levels by quantitative reverse transcription-PCR (qRT-PCR) in plasmid-free PAOU and PAO1 strains at 20 h of growth. The *rhlG* mRNAs were 3-fold less abundant in PAOU than in the wildtype strain PAO1 (Additional file 1: Figure S1, Expression levels of rhlG gene). These results confirmed the involvement of AlgU in *rhlG* transcription, in agreement with the sequence of the novel promoter identified by our 5′-RACE PCR experiment.

We did not identify the transcription start site at position −65 (Figure 1) resulting from a σ^54^-dependent promoter [4]. To rule out the involvement of σ^54^ in our strain and conditions, we used the *prrhlG*::*luxCDABE* fusion in *P. aeruginosa* PAO6358, which was constructed from PAO1 by deleting a large part of the *rpoN* gene encoding σ^54^[24]. The luminescence was 1.7 to 7 fold lower in *P. aeruginosa* PAO6358 than in PAO1 from 8 to 30 h of growth (Figure 2B), indicating that σ^54^ plays indeed an important role in *rhlG* transcription. This was furthermore confirmed by qRT-PCR, which showed that *rhlG* mRNAs were 5-fold less abundant in PAO6358 than in PAO1 at 20 h of growth in PPGAS (Additional file 1: Figure S1). Altogether, three promoters, each dependent on a distinct sigma factor (σ^70^, AlgU and σ^54^), are thus involved in *rhlG* transcription.

### The quorum sensing signal molecule C_4_-HSL inhibits *rhlG* transcription

Since the putative “lux box” found in the *rhlG* promoter region (Figure 1) was proposed to be the binding site of the quorum sensing regulator RhlR [9], we examined the *prrhlG* activity in *P. aeruginosa* PDO100 strain in which the *rhlI* gene is inactivated [25]. This gene encodes the RhlI enzyme responsible for the synthesis of C_4_-HSL which activates RhlR. The *prrhlG*::*luxCDABE* fusion led to luminescence values about 1.6-fold higher in *P. aeruginosa* PDO100 than in PAO1 during stationary phase (Figure 2C), ie when C_4_-HSL accumulates to high concentrations in culture medium [18]. Consistently, the *rhlG* mRNA level assayed by qRT-PCR was 2.6-fold fold higher in PDO100 than in PAO1 at 20 h of growth (Additional file 1: Figure S1). These results were surprising since they indicated that the *prrhlG* activity was inhibited by the Rhl QS system. To further investigate this point, we first added C_4_-HSL at a final concentration of 10 μM to the PPGAS medium when inoculating *P. aeruginosa* PDO100(pAB134). This led to luminescence levels similar to those of PAO1(pAB134) (Figure 2C), confirming that C_4_-HSL has a negative effect on the *prrhlG* activity.

### *prrhlG* activity is induced under hyperosmotic stress

**Figure 3:**
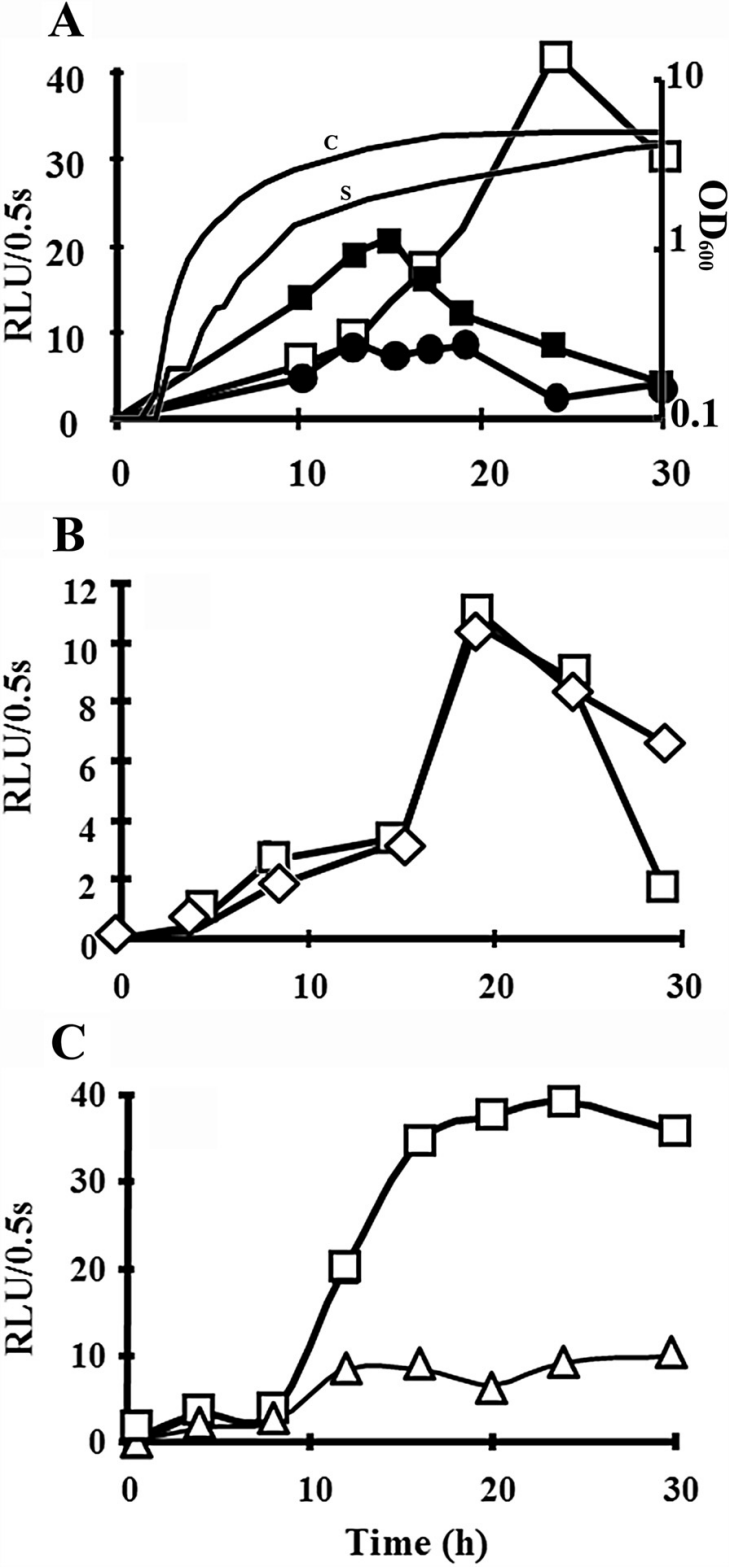
**Transcriptional activity of *rhlG *under hyperosmotic stress.** Promoter activity was followed by measuring luminescence from strains harbouring pAB134, which contain *rhlG::luxCDABE* transcriptional fusion. Activity was measured in *P. aeruginosa* PAO1 wildtype with or without NaCl (respectively white and black squares) and supplemented with 1 mM GB in presence of NaCl (black circles) **(A)**. Hyperosmotic stress effect on *rhlG* activity was followed in PA6358 (*rpoN* mutant, diamonds) compared to wildtype (squares) during the same set of experiments **(B)**. Hyperosmotic stress effect on *prrhlG* activity was followed in PAOU (*algU* mutant, triangles) compared to wildtype (squares) during the same set of experiments **(C)**. Activity is expressed in Relative Units of Luminescence per 0.5 second in function of time growth. Gain for luminescence detection was automatically set for each experiment. Results are representative of 2 complete experiments and of several additional experiments with fewer time points, standard deviations were < 7% for all values. Curve without symbol in panel A: growth curve of PAO1 with 0.5 M NaCl (S) or not (C).

We previously showed that hyperosmotic stress (0.5 M NaCl in PLM63 or PPGAS medium) abolishes rhamnolipid production and inhibits the transcription of genes involved in rhamnolipid synthesis (*rhlAB, rhlC*) and in C4-HSL synthesis (*rhlI*) [17,18]. In PPGAS culture, we observed by qRT-PCR performed on the same mRNA extraction as in [18] that the amount of *rhlG* mRNA was 3.7-fold higher after 20 h of growth in hyperosmotic condition (0.5 M NaCl in PPGAS medium) (Additional file 1: Figure S1). This observation was confirmed using the *prrhlG::luxCDABE* fusion: the luminescence indeed increased until 24 h of growth in hyperosmotic condition, while it decreased in the absence of NaCl from 16 h (Figure 3A). The delay in luminescence increase observed in the presence of NaCl probably corresponded to the growth lag due to the hyperosmotic condition (Figure 3A). We previously observed that the presence of the osmoprotectant glycine betaine during hyperosmotic stress in PPGAS medium did not improve growth, but at least partially prevented the down-regulation of *rhlAB*, *rhlC*, and *rhlI* genes and partially restored rhamnolipid production [18]. Similarly, glycine betaine prevented the increase of *prrhlG* activity under hyperosmotic stress, the *prrhlG* activity being even lower in the presence of 0.5 M NaCl and glycine betaine than in regular PPGAS (Figure 3A).

To determine which of the *rhlG* promoters is responsible for this response to hyperosmotic condition, we used the PAO6358 (RpoN mutant) and PAOU (AlgU mutant) strains. No significant difference was observed when comparing the *prrhlG* activity in the PAO1 and PAO6358 strains, showing that σ^54^ is not involved in *prrhlG* induction in hyperosmotic condition (Figure 3B). On the opposite, the *prrhlG* activity remained low under hyperosmotic stress in the PAOU mutant (Figure 3C), showing that AlgU is responsible for increasing the *rhlG* transcription in this environmental condition. qRT-PCR assays confirmed this result, since we observed a 3.7 fold increase in *rhlG* mRNA level after 20 h of growth under hyperosmotic condition in PAO1, but not in PAOU (Additional file 1: Figure S1).

### Rhamnolipid and PQS productions are not altered in a *rhlG* mutant

Since data from Campos-Garcia *et al.*[4] and from Zhu and Rock [3] were discordant, and since our data showed that *rhlG* is not coordinately regulated with the other genes involved in biosurfactant biosynthesis (*rhlAB*, *rhlC*), we constructed our own *rhlG* mutant (PAOGAB) of PAO1 in order to clarify the RhlG involvement in rhamnolipid production. Rhamnolipids produced by the strains were then quantified both intra- and extra-cellularly. In PAOGAB compared to PAO1, we observed a slight decrease (~20%) of extra-cellular production that complementation by *rhlG* did not restore. No difference at all was observed in the intracellular fraction (Additional file 1: Figure S2, Extracellular and intracellular production of di-rhamnolipid). Our results were thus concordant with [3], but discordant from [4] where rhamnolipid production was totally suppressed. The ACP5 mutant used in [4] was constructed by inserting a tetracycline resistance cassette within *rhlG*, which could have a polar effect on the expression of the downstream gene, PA3388. Our PAOGAB mutant was constructed using a *cre*-*lox* system which allows the construction of deletion mutant without antibiotic resistance gene to avoid altering the expression of downstream gene(s) [26]. We suspected that Campos-Garcia *et al.* observations could result from a defective expression of PA3388, or of both *rhlG* and PA3388. We therefore constructed a PA3388 single deletion mutant and a double *rhlG/*PA3388 mutant. These two mutants displayed similar levels of rhamnolipid production as the PAOGAB and PAO1 strains (Additional file 1: Figure S1), showing that neither *rhlG* nor PA3388 is involved in rhamnolipid biosynthesis.

Since β-ketoacyl-ACP, a potential substrate of RhlG, is a precursor for both rhamnolipid and PQS biosynthesis [4,27], we further examined PQS production, but no significant difference was observed between PAO1 and PAOGAB (data not shown).

## Discussion

Although rhamnolipid production is well described in *P. aeruginosa,* only few reports investigated the involvement of *rhlG* in this biosynthesis pathway. We focused our study on transcriptional regulation. A previous study [4] identified two sigma factors involved in *rhlG* transcription, σ^70^ and σ^54^. Promoter mapping led us to discover an additional promoter and a third sigma factor involved: AlgU. Since *rhlG* has been found to be involved in rhamnolipid production [4], and since the authors described a “lux box” potentially recognized by RhlR/C_4_-HSL, it was suggested that *rhlG* was regulated similarly as the other genes involved in the rhamnolipid biosynthesis (*rhlAB* and *rhlC*). Here we found that it was not the case. Whereas C4-HSL is required for *rhlAB* transcription [10], we observed that it has a negative effect on *rhlG* promoter activity. The “lux box” overlaps the AlgU-dependent promoter (Figure 1) and it is possible that the binding of RhlR/C_4_-HSL onto the “lux box” prevents the activity of this promoter. In support of this hypothesis, transcriptional fusions showed that AlgU is the main sigma factor for *rhlG* transcription during stationary phase (from about 16 h of culture) (Figure 2A and B), when C_4_-HSL reaches its maximal concentration [17,18]. We also observed that *rhlG* promoter activity and mRNA level were increased under hyperosmotic stress conditions. This result is in agreement with the above hypothesis since C_4_-HSL production is reduced under hyperosmotic stress [18], whereas AlgU activity is induced in this condition [28]. We confirmed that the increase of *rhlG* promoter activity under hyperosmotic stress was dependent on AlgU but not on σ^54^. By contrast, *rhlAB* and *rhlC* mRNA levels were reported to be lower under osmotic stress and rhamnolipid production was abolished [17,18]. It should be noted that the “lux box” found in *rhlG* promoter region (Figure 1) does not match exactly the consensus (the most conserved motif is CT-N12-AG [29], whereas CT and AG are separated by 13 nucleotides upstream of *rhlG*) and is closely related neither to an *rhl*-responsive nor to a *las*-specific binding sequence as defined in [30]. The consequence of such an unusual “lux box” is unknown, but we cannot exclude that this sequence is actually not a RhlR binding site and that RhlR/C_4_-HSL acts indirectly on *rhlG* transcription, for example by inducing the expressing of a gene encoding an unknown *rhlG* repressor.

Consistently with the inverse regulation of *rhlG* and the genes involved in rhamnolipid synthesis, rhamnolipid production was not dramatically impaired in the *rhlG* null mutant that we constructed in *P. aeruginosa* PAO1, in agreement with Zhu and Rock [3] data. This raises the question of the RhlG function. RhlG was confirmed to be an NADPH-dependent β-ketoacyl reductase, but its substrates are not carried by the ACP [6]. Since we observed an increase of *rhlG* transcription under hyperosmotic stress, we examined if *rhlG* was involved in osmotic stress response, but no difference was observed in terms of growth and survival between the *rhlG* mutant and its parental PAO1 strain after osmotic stress (data not shown). We furthermore tested a number of phenotypes related to rhamnolipids production (PQS production, motility [swarming, twitching, swimming], biofilm formation in flow cell chamber), but the *rhlG* mutant displayed no difference compared to PAO1 (biofilms are shown in Additional file 1: Figure S3, CLSM images of biofilms). Since *rhlG* likely forms an operon with the PA3388 gene of unknown function [4], we furthermore constructed the single PA3388 mutant and the double *rhlG*/PA3388 mutant. They both failed to display a phenotype related to rhamnolipid production or to any of the other tested characteristics (additional file).

## Conclusions

We present here the first detailed study of *rhlG* transcription, revealing a complex regulation since it relies on three sigma factors and is negatively affected by cell-to-cell communication molecule C_4_-HSL. *rhlG* transcription is induced by hyperosmotic stress via the ECF sigma factor AlgU and inversely regulated compared to the genes involved in rhamnolipid synthesis. Finally, we definitely ruled out that neither *rhlG* nor the downstream PA3388 gene are required for rhamnolipid production, but we failed to identify a function in which these genes are involved.

## Methods

### Bacterial strains and culture conditions

Strains and plasmids are listed in Table 1. Cultures were performed in LB (NaCl 10 g.l^−1^; yeast extract 5 g.l^−1^; tryptone 10 g.l^−1^) and in PPGAS (NH_4_Cl 20 mM; KCl 20 mM; Tris–HCl 120 mM; MgSO_4_ 1.6 mM; glucose 0.5%; tryptone 1%, adjusted to pH 7.2 [19]) media at 37°C with shaking, and growth was followed by measuring optical density at 600 nm (OD600). Solid media were LB agar or *Pseudomonas* isolation agar (PIA) (Gibco-BRL, Grand Island, N.Y.). Hyperosmotic conditions were obtained by including 0.5 M NaCl into the medium before inoculation. Glycine betaine (GB) (Sigma-Aldrich Co., l’Isle d’Abeau Chesnes, France) was used at a final concentration of 1 mM. When indicated, C4-HSL (Sigma-Aldrich Co.) was added at a final concentration of 10 μM. Antibiotics were used at the following concentrations when necessary. For *E. coli*: 50 μg.ml^−1^ kanamycin (Km), 35 μg.ml^−1^ gentamycin (Gm), 100 μg.ml^−1^ ampicillin (Amp), and 10 μg.ml^−1^ tetracyclin (Tc); and for *P. aeruginosa*: 400 μg.ml^−1^ Gm, 600 μg.ml^−1^ carbenicillin (Cb), and 150 μg.ml^−1^ Tc.

**Table 1:** Bacterial strains and plasmids used in this study

**Strain or plasmid**	**Description**	**Reference(s) or source**
*P. aeruginosa*		
PAO1	Plasmid-free strain	[31]
PAO6358	*rpoN* mutant	[24]
PDO100	*rhlI* mutant	[25]
PAOGAB	*rhlG* mutant	This study
PAOFDO	PA3388 mutant	This study
PAOJBB	*rhlG*/PA3388 mutant	This study
PAOU	*algU* mutant	[21]
*Escherichia coli*		
Top10	Electrocompetent cells	Invitrogen
S17.1	*RecA pro* (RP4-2Tet::Mu Kan::Tn7)	[26]
		
**Plasmids**		
pBBR1MCS-5	Cloning vector, Gm^R^	[32]
pAB133	Promoter-less *luxCDABE* operon cloned in pBBR1MCS-5, Gm^R^	[17]
pAB134	*rhlG* promoter cloned in pAB133, Gm^R^	This study
pEX100Tlink	*P. aeruginosa* suicide vector, Amp^R^	[26]
pUCGm*lox*	Amp^R^, Gm^R^, pUC18-based vector containing the *lox* flanked *aacC1*	[26]
pCM157	*cre* expression vector, Tc^R^	[33]
pGAB10	Deleted *rhlG* cloned in pEX100Tlink, Amp^R^	This study
pFAB1	Deleted PA3388 cloned in pEX100Tlink, Amp^R^	This study
pJBB1	Deleted *rhlG*-PA3388 operon cloned in pEX100Tlink, Amp^R^	This study
pGAB10.14	*lox* flanked *aacC1* from pUCGm*lox* cloned in pGAB10, Amp^R^ Gm^R^	This study
PFAB1.13	*lox* flanked *aacC1* from pUCGm*lox* cloned in pFAB1, Amp^R^ Gm^R^	This study
pJBB11	*lox* flanked *aacC1* from pUCGm*lox* cloned in pJBB, Amp^R^ Gm^R^	This study
pGAB	Complementation, *rhlG* cloned in pBBR1MCS-5, Gm^R^	This study

### Rhamnolipid and PQS analyses

PQS and the major rhamnolipid species (di-rhamnolipid Rha–Rha–C10–C10) were identified and quantified from culture supernatants and cellular pellet using LC-MS as previously reported [17,18].

### Biofilm formation

Biofilms were grown for 24 h in flow cell chambers under dynamic conditions (2.5 ml.h^−1^ of LB medium) at 37°C as previously described [21], stained with 5 μM SYTO 9 green (Molecular Probes, Invitrogen), observed and quantified by Confocal Laser Scanning Microscopy (CLSM) with a TCS-SP2 microscope (Leica Microsystems, Heidelberg, Germany) using a 63x oil immersion objective.

### Bioluminescence assays

Induction of bioluminescence in bacteria carrying *luxCDABE* reporter plasmids was detected in optiplateTM 96 wells using the Lumicount apparatus (PerkinElmer, Boston, Ma.), with a gain set at 1 or 6 and with photomultiplier tubes (PMT) set at 1100. 100 μl of bacterial suspensions were adjusted to the lowest optical density of the different samples, and bioluminescence values of a negative control strain (containing pAB133) were subtracted from values resulting from pAB134-containing strain(s) [34]. Bioluminescence was expressed in RLU/0.5 s.

### mRNA quantification by quantitative reverse transcription-PCR (qRT-PCR)

RNAs were extracted using RNA protect bacteria reagent, RNeasy Midi Kit, and RNase-Free DNase Set (Qiagen, Hilden, Germany). RNAs were converted to cDNAs using the High Capacity cDNA Archive Kit (Applied Biosystems, Foster City, Ca.). *rhlG* mRNAs were quantified by real-time PCR amplification of their cDNAs with the 7300 Real Time PCR System apparatus and SYBR Green PCR Master Mix (Applied Biosystems), using procedures previously described [21] and the primers shown in Table 2.

**Table 2:** Oligonucleotides used in this study

**Name**	**5′-3′ sequence**^**a**^	**Used for**	**References**
prRhlG1	attat*gagctc*CATCCTGTTCGTCCTGTTC (*Sac*I)	cloning of *rhlG* promoter	This study
prRhlG2	atatt*actagt*GGGAGACCAGCCTACGAT (*Spe*I)	cloning of *rhlG* promoter	This study
rhlG33	GGATGCTGGCGAAGGAACT	qRT-PCR	This study
rhlG34	GTCATGCGGCTCGGAAAG	qRT-PCR	This study
16sFad1	CAGGATTAGATACCCTGGTAGTCCAC	qRT-PCR	[35]
16sRad2	GACTTAACCCAACATCTCACGACAC	qRT-PCR	[35]
rhlGko1	tata*gaaTTC*GTCGAGCACTACCTGTTG (*Eco*RI)	Knock out	This study
rhlGko2	tata*ctGCAG*TTGCTGGATGCAGGA (*Pst*I)	Knock out	This study
rhlGko3	tata*ctgcaG*CCTACATGACCGGCAAC (*Pst*I)	Knock out	This study
rhlGko4	atat*aagcTT*GGTCGAGCCGCTGAT (*Hin*dIII)	Knock out	This study
PA3388ko1	tata*gaaTTC*ATCTGCGCACGTGAC (*Eco*RI)	Knock out	This study
PA3388ko2	tata*tctAGA*AACGCTGTGGGTCATG (*Xba*I)	Knock out	This study
PA3388ko3	ttat*tctaGA*TATCAAGCCCTACGTACCCTAC (*Xba*I)	Knock out	This study
PA3388ko4	attt*aagcTT*CCGTGTACTGCATCTTTATCA (*Hin*dIII)	Knock out	This study
PA3388ko5	ttatt*ctgcaG*ATATCAAGCCCTACGTACCCTAC (*PstI*)	Knock out	This study
Gsp1G	TGCGTCTTGAGTATTCTTCA	5′-RACE PCR	This study
Gsp2G	GCCCTACCGTATAGAGAAAA	5′-RACE PCR	This study
NesG	CCGTAATTCGTTATTTCCAT	5′-RACE PCR	This study

^a^Capital bases are complementary to the target sequence and italic sequences correspond to the restriction sites indicated in brackets.

### Nucleic acid procedures

Restriction enzymes, T4 DNA ligase, and alkaline phosphatase were purchased from Invitrogen (Carlsbad, Ca., USA). PCR reactions were performed using the FailsafeTM PCR reagent with 2x Premix D (Epicentre Biotechnologies, Madison, Wi., USA). Plasmids and RNAs were purified using the QIAprep Spin Miniprep Kit and RNeasy Midi Kit (Qiagen). *E. coli* (commercial electrocompetent Top10 [Invitrogen] or S17.1 cells) and *P. aeruginosa* were transformed by electroporation as described by manufacturer and in [36], respectively. For mutagenesis experiments, *P. aeruginosa* was transformed by conjugation [21].

### Construction of reporter plasmids carrying the *rhlG* promoter region

The transcriptional fusion between the *rhlG* promoter region (*prrhlG*) and the *luxCDABE* reporter operon was constructed as follows. The DNA fragment containing *prrhlG* was amplified from *P. aeruginosa* PAO1 chromosomal DNA by PCR with the prRhlG1 and prRhlG2 primers (Table 2). The PCR product was digested with *Sac*I and *Spe*I and inserted into *Sac*I-*Spe*I-digested pAB133 [17], yielding pAB134 (Table 1).

### Promoter mapping by 5′-RACE PCR

Total RNAs were isolated from *P. aeruginosa* PAO1 grown in PPGAS medium using the MasterPure RNA Purification kit (Epicentre Biotechnologies). The 5′ end of *rhlG* mRNAs was amplified using the 5′-RACE System for Rapid Amplification of cDNA Ends, Version 2.0 (Invitrogen, Paisley, UK) according to the manufacturer’s instructions. The primers used for cDNA synthesis, and for the first and second PCR reactions are listed in Table 2. The final PCR products of 5′-RACE amplifications were then sequenced (Cogenics, Takeley, UK).

### Gene inactivation

Mutants of *P. aeruginosa* PAO1 were obtained by allelic exchange as previously described [21]. The flanking regions of the gene to delete (*rhlG* or PA3388) were PCR-amplified with primer pairs rhlGko1/2 and rhlGko3/4 or PA3388ko1/2 and PA3388ko3/4 (Table 2), joined (1/2 with 3/4) and cloned in pEX100Tlink, yielding pGAB10 and pFAB1 (Table 1), respectively. To delete both *rhlG* and PA3388 genes, the DNA fragments amplified with primer pairs rhlGko1/2 and PA3388ko5/4 (Table 2) were joined and cloned in pEX100Tlink, yielding pJBB (Table 1). The *lox*-*aacC1*-*lox* cassette of pUCGmlox was then subcloned in-between the two joined PCR fragments of pGAB10, pFAB1, and pJBB1, leading to pGAB10.14, pFAB1.13 and pJBB11, respectively (Table 1). The latter plasmids were introduced into the *E. coli* donor/helper strain S17.1, from which they were transferred by conjugation into *P. aeruginosa* PAO1. After recombination and *aacC1* excision by the pCM157-encoded Cre recombinase, an internal deletion of 343 pb, 371 pb and 831 pb was obtained for *rhlG*, PA3388, and *rhlG*/PA3388, respectively. After verification by PCR and sequencing, the resulting strains selected for further studies were named PAOGAB, PAOFDO and PAOJBB (*rhlG*, PA3388 and *rhlG*/PA3388 mutants, respectively) (Table 1).

To complement the *rhlG* mutation, the DNA fragment including *rhlG* and its promoter region was amplified by PCR using the primers prRhlG1 and rhlGko4 (Table 2). The amplicon was inserted into pBBR1MCS-5, yielding pGAB plasmid (Table 1).

## Competing interests

The authors declare that they have no competing interests.

## Authors’ contributions

AB performed all the experiments and co-drafted the manuscript. AD supervised the study and co-drafted the manuscript. Both authors read and approved the final manuscript.

## Supplementary Material

Additional file 1: Figure S1 Expression levels of rhlG gene. **Figure S2.** Extracellular and intracellular production of di-rhamnolipid. **Figure S3.** CLSM images of biofilms. Click here for file
