# Supplementary material for: The Pseudomonas aeruginosa rhlG and rhlAB genes are inversely regulated and RhlG is not required for rhamnolipid synthesis
Source: BMC Microbiol. 2014 Jun 19;14:160. doi: 10.1186/1471-2180-14-160 (PMC4074388; doi:10.1186/1471-2180-14-160)
Supplement: Additional file 1: Figure S1 — Expression levels of rhlG gene. Figure S2. Extracellular and intracellular production of di-rhamnolipid. Figure S3. CLSM images of biofilms. [file 1471-2180-14-160-S1.pdf]

The *Pseudomonas aeruginosa rhlG* and *rhlAB* genes are inversely regulated and RhlG is not required for rhamnolipid synthesis.

Alexis BAZIRE and Alain DUFOUR

Additional data

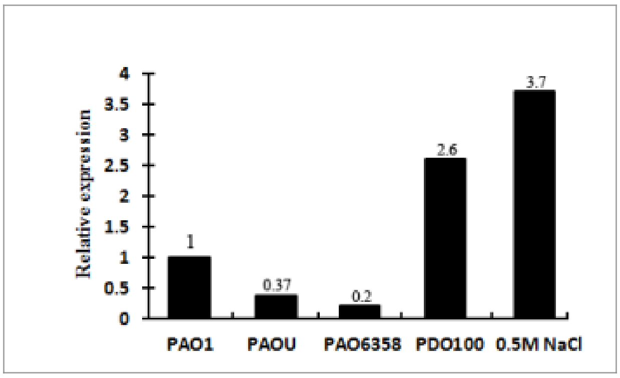

Figure S1. Expression levels of *rhlG* gene in the *algU* mutant PAOU, *rpoN* mutant PAO6358, *rhlI* mutant PDO100 relative to those in PAO1, and osmotic stress (0.5 M NaCl) effect on *rhlG* expression level in PAO1 relative to the level in PAO1 without NaCl. RNAs were extracted after 20 h of growth in liquid PPGAS medium supplemented or not with 0.5M NaCl and were assayed by qRT-PCR. A relative value of <1 indicates a reduction of gene expression and >1 indicates an increase. Values are indicated at the top of each bar. 16S rRNA was used as an endogenous control to normalize the RNA input and reverse transcription efficiency. PCRs were performed in triplicate, and the standard deviations were < 0.15 C_T. The values are the means for at least two independent experiments.

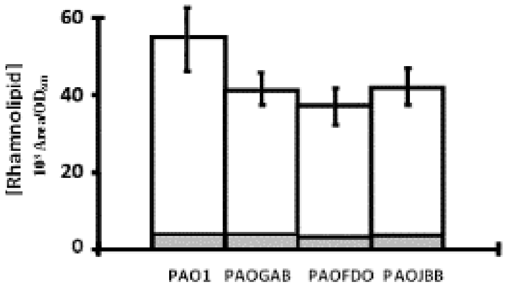

Figure S2. Extracellular (white bars) and intracellular (grey bars) production of di-rhamnolipid Rha–Rha–C10–C10 (ion m/z 649) in PAO1, PAOGAB (*rhlG* mutant), PAOFDO (*rcsF* mutant) and PAOJBB (*rhlG/rcsF* mutant). Rhamnolipid concentration was shown as the liquid chromatography–mass spectrometry peak surface areas divided by the OD_{600} values of the cultures (10^3 Area/OD_{600}). The values are averages from at least three experiments, with the standard deviations indicated by the error bars.

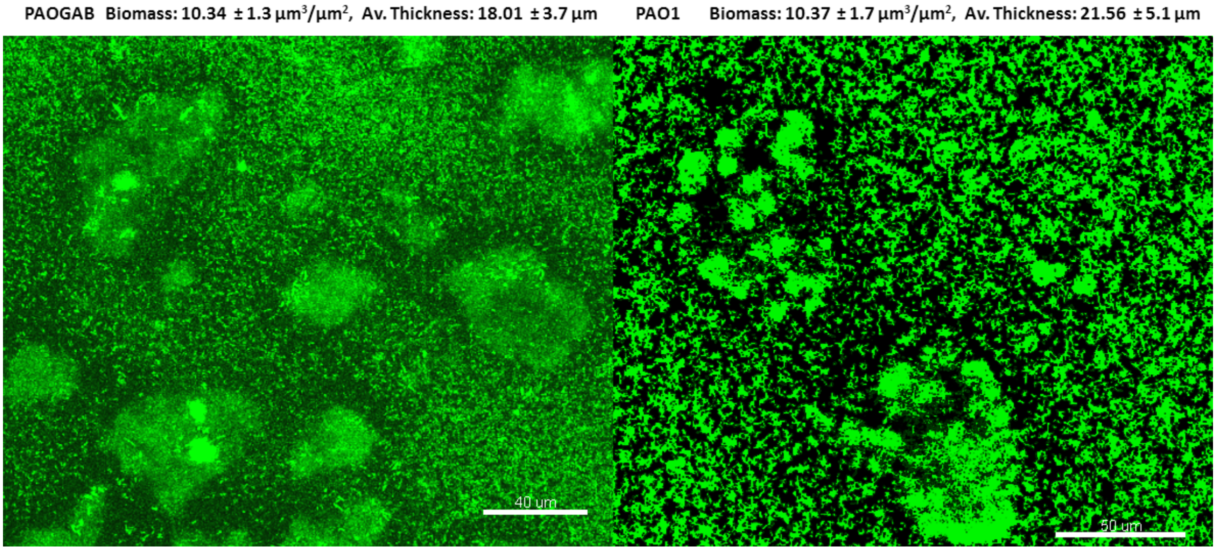

Figure S3. Confocal Laser Scanning Microscopy images of *Pseudomonas aeruginosa* PAO1 and PAOGAB (*rhlG* mutant) biofilms. Biofilms were grown on glass surfaces at 37°C for 24 h under a flow of LB medium as described in Bazire et *al*., 2010. Biofilms were visualized by staining the cells with Syto 9 green. Biomasses and average thicknesses were calculated with Comstat software (Heydorn A, Nielsen AT, Hentzer M, Sternberg C, Givskov M, Ersboll BK, Molin S. *Microbiology* 2000, **10**:2395-2407). The values are averages from three independent experiments and 3 acquisitions by experiment. Bars, 40 µm (PAOGAB panel) and 50 µm (PAO1 panel).
